# Quality Evaluation of a Checklist for Intubation Preparation in Graduate Medical Education

**DOI:** 10.7759/cureus.25830

**Published:** 2022-06-10

**Authors:** Philip A Pazderka, Joshua Mastenbrook, Joseph Billian, Ryan Caulfield, Fahad Khan, Glenn Ekblad, Micheal Williams, John Hoyle

**Affiliations:** 1 Emergency Medicine, Western Michigan University Homer Stryker M.D. School of Medicine, Kalamazoo, USA; 2 Division of Epidemiology and Biostatistics, Western Michigan University Homer Stryker M.D. School of Medicine, Kalamazoo, USA; 3 Emergency Department, North Colorado Medical Center, Greeley, USA; 4 Emergency Department, Piedmont Henry Hospital, Stockbridge, USA; 5 Emergency Medicine, Salem Veterans Affairs Hospital, Salem, USA; 6 Palliative Care, Borgess Medical Center, Kalamazoo, USA; 7 Emergency Medicine and Pediatrics, Western Michigan University Homer Stryker M.D. School of Medicine, Kalamazoo, USA

**Keywords:** intubation, rapid sequence intubation, quality improvement, education, checklist, endotracheal intubation

## Abstract

Background

Rapid sequence intubation (RSI) is a multistep process that emergency physicians commonly perform. Unfortunately, there is little published in the graduate medical education literature regarding the use of checklists for RSI education.

Methods

We developed a pre-intubation checklist for RSI preparation and evaluated emergency medicine residents’ use of it. We developed the checklist using a three-round modified Delphi process among a group of emergency medicine faculty physicians within our institution. Over a three-year period, residents were randomized into two groups: a “checklist group” and a “without-checklist group.” Residents were then evaluated for RSI critical step completion in a simulated critically ill patient by two independent study investigators. Inter-rater reliability kappa scores were calculated. Following completion of the scenario, residents in both groups were asked to complete an anonymous survey. Both groups had access to the checklist at the time of the survey. The survey was used to determine if they found the checklist helpful. Odds ratios with p-values, at an alpha of 0.05 for significance, were computed for checklist items comparing the checklist and without-checklist groups. Data analysis was performed using SAS software (SAS, Cary, NC v 9.4). This study was approved by the authors’ Institutional Review Board.

Results

Each assessment was completed by two investigators. Inter-rater reliability was substantial (κ=0.79). Residents having access to the checklist were more likely to verbalize a critical step with a p-value of < 0.0001 and an odds ratio of 2.17 (95% CI: 1.48, 3.19). The checklist group normalized vital signs prior to intubation in 25/28 (89%, 95% CI: 72.81, 96.29) versus only 6/29 (21%, 95% CI: 9.85, 38.39) with a p-value of <0.0001 in the without-checklist group. The checklist group evaluated for difficult laryngoscopy 26/28 (93%, 95% CI: 77.36, 98.02) versus only 21/29 (72%, CI 95% 54.28, 85.30) with p=0.0223 in the without-checklist group. All of the surveyed residents indicated that the checklist would be helpful for future use in the ED.

Conclusion

This RSI checklist improved adherence to preparatory steps of RSI. Utilizing a checklist increased evaluation for a difficult airway and normalizing vital signs. Residents found the checklist helpful for ED use.

## Introduction

Emergency medicine residents are expected to manage airway emergencies in the ED. Rapid sequence intubation (RSI) is a complex, multistep process that is carried out on critically ill patients in the ED. This complex process is prone to error [1]. Given the number of steps necessary to perform RSI correctly, it is possible that emergency medicine residents may be unable to remember them all without a cognitive aid. Many EDs have premade intubation kits, which contain essential tools for intubation. However, these kits themselves do not remind the emergency medicine residents of the critical pre-intubation steps. 

Checklists are essential tools that can reduce cognitive load, improve standardization, and ensure that all the steps in a process are completed [2]. Individuals under stress, fatigue, and interruption may omit necessary steps [2,3]. Omissions during preparation for RSI may result in serious patient safety sequelae, including hypoxia, hypotension, aspiration, and bradycardia [4,5].

Checklists have been used in industry and aviation for years [6]. Recently, several studies have developed standardized checklists for intubation [1,3,7]. However, our faculty have observed that preparation for intubation was a non-standardized process and that critical steps, such as assessing for a difficult airway, were frequently omitted. Therefore, we wished to develop an educational instrument that would aid in resident education for RSI. In addition, we hypothesized that the use of a checklist would decrease frequently omitted steps in the RSI process for a non-crashing patient, and the use of such a checklist would educate emergency medicine residents on the multiple steps of intubation.

## Materials and methods

Study design and participants

The setting of this study was a three-year university-based emergency medicine residency program affiliated with two community hospitals. The participants were emergency medicine residents spanning four classes for a total of 57 individuals. The data were collected at the end of the academic year 2017-2018 and the beginning of 2018-2019. The study was approved by the Western Michigan University Homer Stryker M.D. School of Medicine Institutional Review Board with an exemption (WMed-2017-0171).

The intervention in this study was developing and deploying a new checklist for RSI preparation. Our emergency medicine faculty determined that the previously published checklist did not contain all of the items necessary for RSI preparation [8]. The faculty held multiple meetings to develop the initial intubation preparation checklist. A literature search was performed with PubMed using the search terms "intubation" and "checklist." The relevant articles were evaluated for applicability to preparation for RSI. The authors also consulted four primary textbooks [8,9,10,11]. From this information, a preliminary 12-item checklist was developed. A modified Delphi method was then used to develop the checklist further. This was done through a questionnaire to 13 emergency medicine faculty experienced in RSI. Three Delphi rounds were used to develop a consensus on what should be included and deleted from the checklist. It was determined that 60% of faculty had to agree to either keep or remove an item. This process yielded the checklist (Appendix 1). All checklist elements were weighted the same. The checklist is meant to be read line by line in preparation for intubation as a "read-do" checklist.

The checklist was then tested via a simulated patient scenario. Participating emergency medicine residents were divided via a randomization program into two groups: those that would use the checklist and those that would not use the checklist. Each residency class was randomized separately to ensure that the same proportion of each postgraduate year (PGY) level was represented in both groups. Participation was voluntary, and residents completed a consent form before enrollment. Two residents did not participate. These individuals were on the study team. Each study participant received scripted, standardized instructions and was explicitly told to verbalize each step they were thinking about (Appendix 2). The checklist group participant received the checklist and was allowed to study the checklist for two minutes before beginning their preparation for RSI. The checklist was also available for those residents to use during their intubation attempts. The without-checklist group participants did not receive the checklist and were allowed two minutes to prepare for RSI as they usually would.

Both groups were presented with the same patient scenario: a patient with a severe chronic obstructive pulmonary disease with respiratory failure, represented by an Airway Management Trainer (Laerdal Corporation, Wappingers, New York) and all necessary intubation equipment. Each group had a study team member who played the role of a nurse to help manage equipment and administer medications (Appendix 3).

Outcomes

Two study investigators observed each intubation and the tasks completed, using a standardized grading form developed from the checklist. It was not possible to blind the residents or the evaluators to the group assignment, as it was apparent which residents had a checklist during the simulation. The study personnel was not blinded as to the nature of the study. Each emergency medicine resident simulation resulted in two sets of evaluations recording whether each step was demonstrated.

Following the intubation, residents from both groups were given a copy of the checklist to review and completed a voluntary, anonymous survey. The residents were blinded to their intubation performance. The survey instrument used in our study was adapted from the study done by Leighton K et al. [12]. Using a five-point Likert-like scale, the 14 survey items evaluated the residents’ opinions of the checklist.

Statistical analysis

All intubation trial and survey data were entered into a REDCap (Research electronic data capture) form [13]. Data analysis was performed using SAS software (SAS, Cary, NC v 9.4). Kappa statistics were calculated for each checklist item to measure inter-rater agreement. A generalized estimating equation (GEE) was used to model the proportion of checklist steps completed by the two groups, as recorded by the two evaluators. The model considered checklist group assignment and PGY level as potential predictors, using a step-up model selection process based on type-3 estimable function analysis. The results of this model were then interpreted as a "number of steps" through a scaled inverse-logit transformation. An additional posthoc analysis used 22 Chi-squared tests to determine the association between group assignment and successful completion of particular checklist steps. In testing these multiple, positively-dependent test statistics, the false discovery rate was controlled at a level of 0.05 using the Benjamini-Hochberg procedure [14]. The survey responses are reported with means and SDs.

## Results

There were 28 residents randomized to the checklist group and 29 to the without-checklist group. The average Kappa across all checklist items was 0.79. GEE analysis showed that the odds of completing a checklist task were 2.17 (p<0.0001, 95%CI: 1.18-3.19) times greater for residents in the with-checklist group than in the without-checklist group. PGY level was not found to be a significant predictor (p = 0.21) for step completion. The residents in the checklist group completed an average of 22 of 42 steps (54.2%, 95% CI: 18-25), while residents in the without-checklist group completed an average of 14 of 42 steps (33%, 95% CI: 13-15). Both groups performed similarly in preoxygenation, voicing two back-up plans, suction preparation, and confirming waveform or colorimetric end-tidal CO2. Checklist use was not associated with the choice or the correct dosing of either sedative or paralytic (Table 1).

**Table 1:** Steps of the simulation completed for the "with checklist" and "without checklist" groups and p-values from corresponding Chi-squared tests. A significant association is indicated with an asterisk. Succinylcholine contraindications, only applicable when the paralytic is succinylcholine, were not included in the Chi-squared tests.

		With Checklist (N=28)		Without Checklist (N=29)		Chi-squared
Checklist item	Outcome	Freq.	Percent	Freq.	Percent	P-value
Preoxygenate with NRB/BVM/BIPAP for 5 minutes/as much as possible	Completed	28	100%	27	93%	0.1572
Attempt to normalize VS as best possible prior to intubation	Completed	25	89%	6	21%	<0.0001*
Equipment check laryngoscope/glidescope	2 Checkboxes Completed	22	79%	8	28%	0.0001*
Back-up plan	2 Checkboxes Completed	22	79%	16	55%	0.061
Suction (turned on with tubing and tonsil tip, available to right hand)	Completed	26	93%	25	86%	0.4134
Assess for difficult direct laryngoscopy	7 Checkboxes Completed	16	57%	1	3%	<0.0001*
	0 Checkboxes Completed	2	7%	9	31%	0.0223*
Assess for difficult Bag Valve Mask	5 Checkboxes Completed	14	50%	0	0%	<0.0001*
	0 Checkboxes Completed	5	18%	20	69%	0.0001*
Assess for difficult Extraglottic Airway	4 Checkboxes Completed	13	46%	0	0%	<0.0001*
	0 Checkboxes Completed	10	36%	27	93%	<0.0001*
Assess for Difficult Cricothyrotomy	5 Checkboxes Completed	12	43%	0	0%	<0.0001*
	0 Checkboxes Completed	9	32%	25	86%	<0.0001*
Airway Oral and Nasal airway, Tube sizes	6 Checkboxes Completed	7	25%	1	3%	0.0192*
	0 Checkboxes Completed	0	0%	0	0%	
Position evaluated	Completed	21	75%	11	38%	0.0048*
Fentanyl	Yes	12	43%	0	0%	<0.0001*
Sedative choice	Ketamine	10	36%	6	21%	0.2367
	Etomidate	18	64%	22	79%	
Sedative dose within the range	Correct	22	79%	23	79%	0.9455
Paralytic choice	Succinylcholine	20	71%	15	52%	0.1266
	Rocuronium	8	29%	14	48%	
Succinylcholine Contraindication 1 - burns > 5 days old	Completed	6/20	30%	0/15	0%	
Succinylcholine Contraindication 2 - Spinal cord injury / stroke > 5 days old	Completed	5/20	25%	0/15	0%	
Succinylcholine Contraindication 3 - muscle damage (crush)	Completed	5/20	25%	0/15	0%	
Succinylcholine Contraindication 4 - Neuromuscular disease (MS, ALS, CP)	Completed	5/20	25%	0/15	0%	
Succinylcholine Contraindication 5 - Intraabdominal sepsis > 5 days	Complete	5/20	25%	0/15	0%	
Paralytic dose within the range	Correct	24	86%	21	72%	0.2182
Confirmation: Wave form or colorimetric end tidal CO2	Completed	19	68%	21	72%	0.1413
Plan for post-intubation sedation	Completed	10	36%	1	3%	0.002*

The checklist group was significantly associated with completing more intubation preparation steps. In every case where there was a significant association, the with-checklist group performed better than the without-checklist group. Completion of the multistep assessments for difficult laryngoscopy, bag valve mask, extraglottic airway, and oral/nasal airway tube sizes all showed significant associations with the checklist group. The checklist group was also significantly more likely to voice normalization of the vital signs before intubation, check for the proper functioning of the laryngoscope, evaluate patient positioning, and plan for post-intubation sedation.

The post-intubation survey showed that both groups of emergency medicine residents would find it helpful to use a checklist during future intubations. They found the checklist to cover the essential preparatory elements for intubation and made them easier to remember. Before this study, residents were unlikely to have used a checklist to prepare for intubation. After the study, residents indicated they were more likely to use a checklist (Table 2).

**Table 2:** Description (mean, SD, sample size) of survey item 5-point Likert responses by checklist group.

	With Checklist			Without Checklist		
Survey Item	Mean	SD	N	Mean	SD	N
Pre-reading assignments prepared me for the airway activity	4.04	0.76	23	4	0.67	27
Briefing before the airway education was beneficial	3.79	0.73	29	4.07	0.87	27
Education on preparation for intubation has increased my confidence.	4.39	0.79	29	4.31	0.6	28
During the simulation, I had the opportunity to practice my preparatory skills.	4.36	0.78	29	4.21	0.77	28
I will use the checklist to help me prepare for intubations in the future.	4.07	0.94	29	4.17	0.66	28
The checklist formatting makes the preparation for intubation easy to remember.	4.11	0.92	29	4.24	0.69	28
I am more confident in my abilities to intubate after this educational exercise	3.75	1.04	29	3.59	0.95	28
Debriefing contributed to my learning	4	0.96	28	3.68	0.9	25
Debriefing was valuable in helping me select the appropriate airway intervention	3.88	0.97	26	3.88	0.86	25
Debriefing provided adequate time to review the critical concepts	4	1	26	3.88	0.95	26
In the past, I have used a checklist before endotracheal intubation on more than 50% of the intubations I carry out.	1.96	1.04	28	2.72	1.28	29
The intubation checklist will be helpful to me in the future.	3.93	0.72	28	4.14	0.69	29
I plan on using the intubation checklist regularly for future intubations.	3.64	0.68	28	4.1	0.82	29
The intubation checklist covered the important preparatory elements for intubation.	4.68	0.55	28	4.34	0.55	29

## Discussion

The use of a checklist by emergency medicine residents during a simulated RSI demonstrated a significant increase in the completion of preparation steps compared to having no checklist. We found there was an increase in evaluation for a difficult airway, assessment of pre-intubation hemodynamics, increase in post-intubation sedation, and proper patient positioning. Such improvements have been shown in other research. In the trauma setting, an ED pre-intubation checklist was associated with a decrease in intubation complications, a reduction in time between paralytics, and confirmed tube placement. It increased adherence to predefined safety measures [15]. Verbally checking a checklist improves team situational awareness and promotes a shared mental model. It lets the team know that the patient may have a difficult airway and compels them to think about potential backup plans. 

Difficult airways are infrequent [16], and an unanticipated difficult airway is a potentially life-threatening occurrence. Unsuccessfully managed airways are associated with increased morbidity and mortality [17]. Recognizing a difficult airway allows physicians to plan, consider the use of different equipment, and develop specific backup plans to avert a failed airway scenario. The checklist group in our study was found to evaluate patient positioning more consistently and to evaluate for difficult airways. As difficult airways are infrequent, it is preferable to predict one and be prepared with a backup plan. Although there was a positive trend when using the checklist, no statistically significant difference was found between the two study groups in terms of voicing a backup plan. The checklist serves as a reminder of these possibilities and aids in mental preparation, mindset, and a shared mental model with the team in case a difficult airway is encountered.

Endotracheal intubation is one of the most crucial tasks in an acutely unstable patient. Pre-intubation hemodynamics are one of the predictive factors for hypotension after intubation [18]. Post-intubation hypotension is associated with increased in-hospital mortality and length of stay [19]. We found that using the checklist was associated with improved attempts to normalize vital signs before intubation. This is a crucial step to avoid post-intubation hypotension and possible cardiac arrest.

Succinylcholine is a depolarizing neuromuscular blocking agent used in RSI since 1951. It has been used safely for many years with practitioners familiar with its adverse effects and contraindications [20]. Physicians need to screen for contraindications before use. We evaluated for six common contraindications to succinylcholine: history of malignant hyperthermia, burns greater than five days, muscle damage greater than five days, spinal cord injury or stroke greater than five days, neuromuscular disease, and intra-abdominal sepsis greater than five days. None of the residents in the "without checklist" group acknowledged all contraindications, while residents in the "with checklist" group performed better. Twenty percent of the checklist group screened for all six contraindications to succinylcholine, versus 0% in the no-checklist group. The checklist serves as a direct reminder to screen for these contraindications before giving the medication.

Post-intubation sedation helps improve ventilator response, ventilator-free days, hospital length of stay, and prevention of ICU delirium. Unfortunately, previous studies have shown that as few as one in four patients receive sedation within 15 minutes of intubation [21]. This presents the possibility that the patient, if conscious, may be paralyzed but not sedated, especially if long-acting paralytics are used. This study shows that using a checklist can increase compliance with post-intubation sedation and serve as a reminder to initiate it.

The post-survey found that few residents use a checklist when preparing for RSI. However, after this educational experience, more residents planned on using the intubation checklist regularly for future intubations. Interestingly, even the residents in the without checklist group were more likely to use a checklist after the event. This may be due to the checklist reminding them how much they may have missed going through the simulated case.

Limitations

There were several limitations to the study. First, we relied on the resident subjects to vocalize the steps they were completing. It is possible they had done this mentally without vocalizing it and would therefore be graded as not completing the step(s). However, both groups were given the same instructions before being tested, and it was stressed to verbalize all steps. It was also noted that some residents would read the checklist and then try to reproduce it by memory. Although these individuals performed better than those in the without-checklist group, they performed worse than those who adhered strictly to the checklist. Therefore, we do not know the full impact of the checklist had it been uniformly utilized as designed. Prior research has shown that even professional airline pilots deviate from checklists [22].

Additionally, checklist training is essential when using a read-do design, with the emphasis that every item must be completed. Accordingly, the checklist is now used in the education of our new residents as an introduction to how to prepare for an RSI during an educational event called "RSI Boot Camp." We have also incorporated checklist use into our annual difficult airway course for all residents. We believe that such spaced repetition is an essential concept in resident education. Future studies will focus on checklist education, enablers and barriers to checklist use, and adherence. Even in the simulated environment, a perceived lack of time was found to be a barrier to use.

## Conclusions

The use of a checklist for RSI preparation resulted in a higher completion of necessary procedural preparation steps, including assessing pre-intubation vital signs, assessing for a difficult airway, having backup plans, and assessing for contraindications to succinylcholine, as performed by emergency medicine residents in a simulated RSI encounter. In addition, the use of this checklist can aid in the standardization of RSI in the ED. The adoption of a standardized checklist will decrease procedural variations and help educate residents in the preparation for RSI.

## Figures and Tables

**Figure 1:**
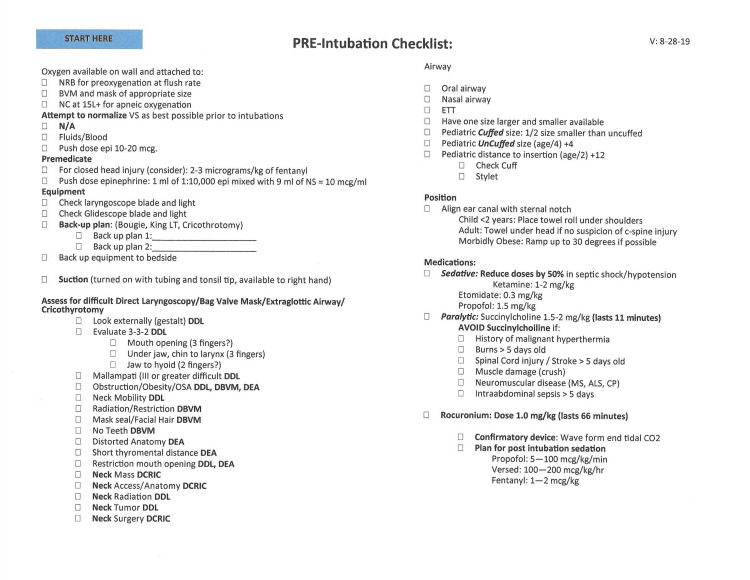
RSI checklist. RSI: Rapid sequence intubation.

## Appendices

Appendix 1

Appendix 2: Participant prompts and nurse actor script

For Intervention Group

Thank you for taking part in our Intubation Study. For this study, we ask that you evaluate the patient, prepare for the intubation, and perform an intubation. Any report of your results will be de-identified in any publication, and your results will be kept confidential.  Do not discuss this scenario with anyone else in order to maintain the integrity of the study.

You are going to be asked to perform RSI on a "patient" represented by a mannequin, using direct laryngoscopy. You have made the decision to intubate the patient because they are showing obvious signs of respiratory failure. For this intubation, you will have 2 minutes to think about what you need prior to the scenario starting. You will also be given a checklist that you must use to help you in your preparation. 

Please use the checklist as a step-by-step guide during your intubation preparation.

Please act as if this is a real case. You must vocalize (say out loud) all of the steps you are taking or even thinking about, such as positioning of the bed at an appropriate height and your pre-intubation assessment. If the evaluator does not understand what you have said, they may ask you to repeat it.

You will have one assistant who will play the role of a nurse. You do not have an RT available. You can ask the nurse to assist you as you normally would.

For this scenario, you will have as many oxygen outlets as you need. We do not have real outlets, so your nurse will "plug them in" for you. Likewise, you will have a suction outlet if needed. Your intubation will conclude when you give one post-intubation breath via BVM.

Case Explanation:

This is a 55-year-old male with significant COPD that has a respiratory failure. He weighs 80 kilograms. You are in a large hospital's ED with usual resources. The patient has no other significant history, and his vitals are pulse 105, BP 120/80, RR: 30, Sat: 89%.

For the Control Group:

Thank you for taking part in our Intubation Study. For this study, we ask that you evaluate the patient, prepare for the intubation, and perform an intubation. Any report of your results will be de-identified in any publication, and your results will be kept confidential.  Do not discuss this scenario with anyone else in order to maintain the integrity of the study.

You are going to be asked to perform RSI on a "patient" represented by a mannequin, using direct laryngoscopy. You have made the decision to intubate the patient because they are showing obvious signs of respiratory failure. For this intubation, you will have 2 minutes to think about what you need prior to the scenario starting.

Please act as if this is a real case. You must vocalize (say out loud) all of the steps you are taking or even thinking about, such as positioning of the bed at an appropriate height and your pre-intubation assessment. If the evaluator does not understand what you have said, they may ask you to repeat it.

You will have one assistant who will play the role of a nurse. You do not have an RT available. You can ask the nurse to assist you as you normally would.

For this scenario, you will have as many oxygen outlets as you need. We do not have real outlets, so your nurse will "plug them in" for you. Likewise, you will have a suction outlet if needed.

Your intubation will conclude when you give one post-intubation breath via BVM.

Appendix 3: Nurse script for intubation study                                          v 4-18-18

Performance Rules for Actor:

1.     You are a nurse.  There is no RT available for this scenario.

2.    At the beginning of the scenario, introduce yourself as listed below in the script.t.

3.     You must stick to the script below.  Do not offer any other information than what is below.

4.     Familiarize yourself with the equipment/supplies that are available (see list) and the drug vials and syringes.

5.     If you are asked for a piece of equipment from the resident, grab it and hand it to them.

6.     If you are asked to draw up a drug, draw up the correct amount in air, do not puncture the vial.

7.     Do Not deliver any medications until the participant tells you to do so.

8.     If you are asked to perform Bag Valve Mask Ventilation on the patient, please do so.

9.     For purpose of the scenario, you will pretend that you have two oxygen outlets.

“Hello, my name is __________, I’m your nurse. I can get whatever equipment or drugs you need." The patient's current vital signs are: Pulse 105   BP: 120/80 RR: 30  Sat: 89% If you are asked for a patient weight: "The patient weighs 80 kilograms". If you are asked to place a non-rebreather, state, "I've placed a non-rebreather." 

If you are asked to place a non-rebreather at flush rate, state, “I’ve placed a non-rebreather and it is running at flush rate.”

If you are asked to place a nasal cannula, place the nasal cannula and state, “I’ve placed the nasal cannula.”

If asked to place a nasal cannula at flush rate, state, “I’ve placed the nasal cannula and it is running at flush rate.”

If you are asked to deliver a medication (i.e. ketamine, etomidate, rocuronium, succinylcholine (sux)), state, “I’m injecting _______ mg of drug _______”.

If you are asked to provide suction, state, “Here is the suction and it is turned on.”

If you are asked to provide an endotracheal tube (ETT tube), hand the size of the tube asked for (7.0, 7.5 or 8.0) and state, “I have checked the cuff and it is good”. DO NOT put a stylet in the endotracheal tube unless specifically asked to.

If asked to put end tidal on the endotracheal tube after the tube is placed, place the end tidal detector on the endotracheal tube.
